# A Practical Nomogram and Risk Stratification System Predicting the Cancer-Specific Survival for Patients With Advanced Hepatocellular Carcinoma

**DOI:** 10.3389/fonc.2022.914192

**Published:** 2022-07-12

**Authors:** Dashuai Yang, Yang Su, Fangrui Zhao, Chen Chen, Kailiang Zhao, Xiangyun Xiong, Youming Ding

**Affiliations:** ^1^ Department of Hepatobiliary Surgery, Renmin Hospital of Wuhan University, Wuhan, China; ^2^ Department of Oncology, Renmin Hospital of Wuhan University, Wuhan, China

**Keywords:** advanced hepatocellular carcinoma, nomogram, cancer-specific survival, risk stratification, AJCC (TNM) staging system

## Abstract

**Background:**

Hepatocellular carcinoma (HCC) has the highest cancer-related mortality rate. This study aims to create a nomogram to predict the cancer-specific survival (CSS) in patients with advanced hepatocellular carcinoma.

**Methods:**

Patients diagnosed with advanced HCC (AJCC stage III and IV) during 1975 to 2018 were obtained from the Surveillance, Epidemiology, and End Results (SEER) database. Qualified patents were randomized into training cohort and validation cohort at a ratio of 7:3. The results of univariate and multivariate Cox regression analyses were used to construct the nomogram. Consistency index (C-index), area under the time-dependent receiver operating characteristic (ROC) curve [time-dependent area under the curve (AUC)], and calibration plots were used to identify and calibrate the nomogram. The net reclassification index (NRI), integrated discrimination improvement (IDI), and C-index, and decision curve analysis DCA were adopted to compare the nomogram’s clinical utility with the AJCC criteria.

**Results:**

The 3,103 patients with advanced hepatocellular carcinoma were selected (the training cohort: 2,175 patients and the validation cohort: 928 patients). The C-index in both training cohort and validation cohort were greater than 0.7. The AUC for ROC in the training cohort was 0.781, 0.771, and 0.791 at 1, 2, and 3 years CSS, respectively. Calibration plots showed good consistency between actual observations and the 1-, 2-, and 3-year CSS predicted by the nomogram. The 1-, 2-, and 3-year NRI were 0.77, 0.46, and 0.48, respectively. The 1-, 2-, and 3-year IDI values were 0.16, 0.15, and 0.12 (*P* < 0.001), respectively. DCA curves in both the training and validation cohorts demonstrated that the nomogram showed better predicted 1-, 2-, and 3-year CSS probabilities than AJCC criteria.

**Conclusions:**

This study established a practical nomogram for predicting CSS in patients with advanced HCC and a risk stratification system that provided an applicable tool for clinical management.

## Introduction

Hepatocellular carcinoma (HCC) is the most common cause of cancer-related death and its incidence rate is increasing (1, 2). According to statistics, HCC accounts for 70–80% of the total burdens of liver disease (3, 4). Although diagnostic techniques for HCC have improved, only 20–35% of patients are diagnosed at an early stage (5), which meant approximately 80% of patients are detected at advanced stage (6). Extensive research results have reported that 5-year survival rate for patients with early-stage HCC can exceed 60% after treatment with tumor resection or liver transplantation (7, 8). Unfortunately, patients with advanced HCC (AJCC Stage III and IV) have been lost the opportunity of surgery, and the 5-year survival rate is only 10% after chemotherapy, radiotherapy, or other local treatment (9, 10). The low rate of early diagnosis and poor prognosis in advanced stage highlight the role of personalized treatment for patients with advanced HCC.

The prognostic models for early-stage HCC have been constructed and validated in several studies (11, 12). However, there is no predictive model for patients with advanced HCC. In the recent years, clinical models based on nomogram have been applied widely for survival prediction of oncology patients due to its advantages of intuitiveness and simplicity (13–15). Such new models can not only effectively promote personalized medicine, but also facilitate clinicians to utilize them for prognosis prediction. In this study, the purpose was to establish a nomogram with new risk stratification system for predicting the prognosis for patients with advanced HCC based on Surveillance, Epidemiology, and End Results (SEER) database.

## Methods

### Material

Patients enrolled in this study were extracted from the SEER18 registry database (1975–2018) by SEER*Stat 8.3.9.2 software for clinical-related data (including baseline demographics, tumor characteristics, therapeutic method, stage at diagnosis, survival status, and survival time) for patients diagnosed with HCC (AJCC Stage III and IV). The SEER database was publicly available and the private data of all patients have been eliminated from the SEER database. Therefore, informed consent and institutional review board approval were not required. The authorization account number for this study was 18419-Nov2020.

### Variables

**Figure 1:**
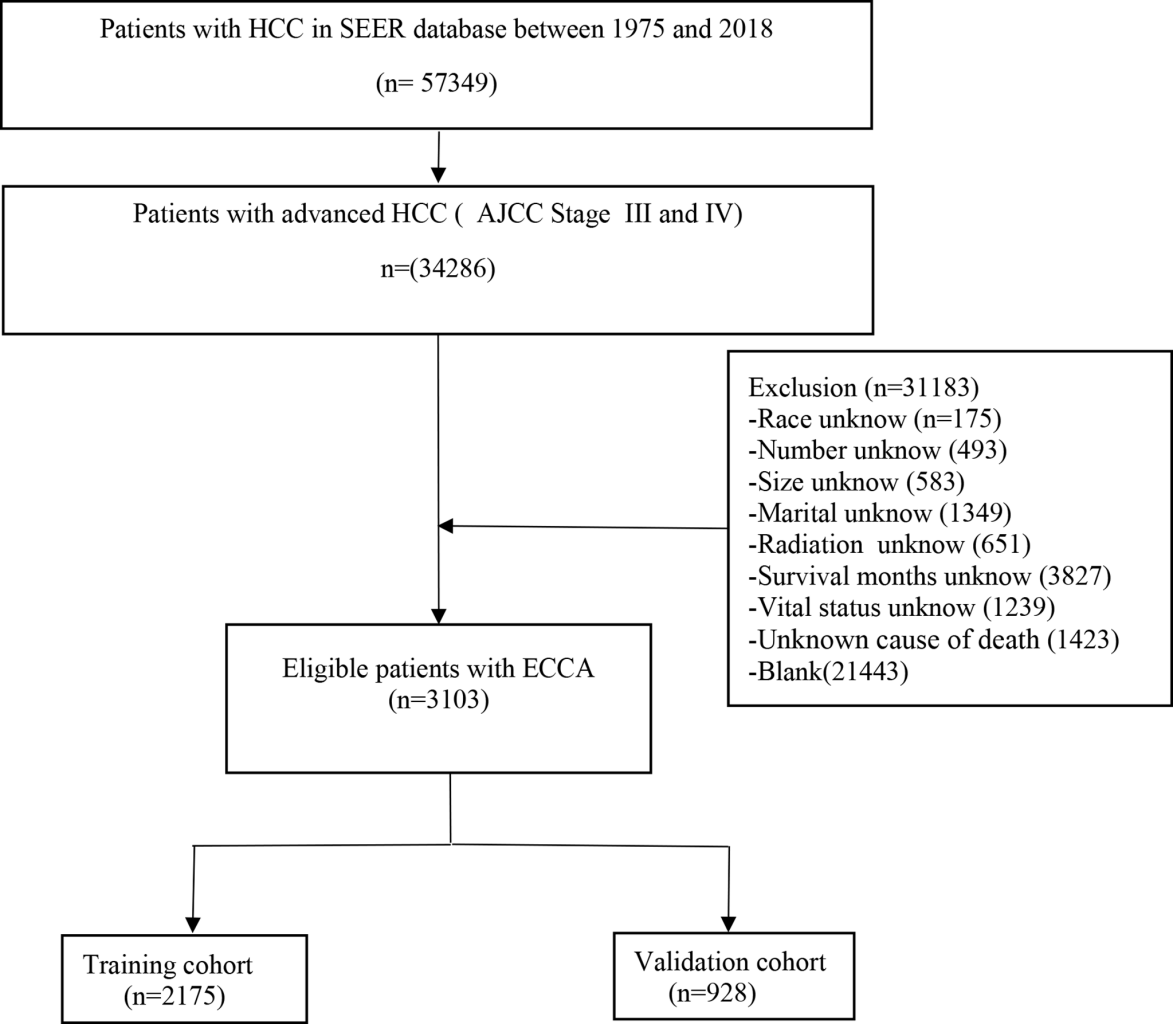
Flow diagram of the advanced hepatocellular carcinoma patients with training and validation cohorts.

Fifteen variables were included in our study (age, gender, race, tumor size, tumor number, AJCC stage, bone metastasis, lymph node metastasis, lung metastasis, treatment, radiation therapy, chemotherapy, marital status, survival months, and survival status). In addition, we adopted the 7th edition AJCC TNM stage system. The inclusion criteria were as follows: (1) diagnosed as advanced HCC; (2) primary tumor location was in the liver; (3) known cause of death; (4) complete treatment information. And the exclusion criteria were as follows: (1) early stage HCC or metastatic liver cancer or other cancers; (2) incomplete information of treatment; (3) death caused by other cancers; and (4) unknown cause of death. The swipe selection process is shown in the flow chart (**Figure 1**).

### Construction and Validation of the Nomogram Model

All patients were randomly divided into two groups at a ratio of 7:3. The training cohort was applied to create the nomogram and the validation cohort was performed for validation. Significant factors (*P* < 0.05), obtaining from univariate and multivariate Cox regression, were performed to construct the nomogram. The consistency index (C-index) and the time-dependent area under the curve (AUC) were calculated by bootstrapping to evaluate discriminative ability. The values of C-index and AUC ranged from 0.5 to 1.0 and were generally divided to low precision (0.5–0.7), moderate precision (0.71–0.90), and high precision (>0.9). The 1-, 2-, and 3-year calibration plots were plotted (1,000 self-help weight samples) to compare the predicted cancer-specific survival (CSS) with that observed in our study, and the 45-degree line was presented as the ideal prediction. DCAs were drawn to estimate the clinical practicality of the nomogram. New risk stratification, which divided patients into low-, middle-, and high-risk groups, was established by X-Tile software basing on the best cutoff value of risk score. Kaplan–Meier curves and log-rank tests were performed to compare the differences of CSS among patients in different risk stratification groups. The C-index, net reclassification index (NRI), integrated discrimination improvement (IDI), and decision curve analysis (DCA) were adopted to evaluate the improvement in predictive capability and effectiveness of the new model.

### Statistical Analysis

SEER*Stat software (version 8.3.9.2) was applied to extract the data and the best cutoff value for the total score were select by X-Tile (version 3.6.1). All data analyses were performed using R software version 4.1.2 (http://www.r-project.org/). The R packages “regplot”, “mstate”, “survival”, “cmprsk”, “hmisc”, “timeROC”, “foreign”, “nricens”, “rmda”, and “DCA” were used to develop and validate the nomogram. All *P* values resulted from the use of two-sided statistical testing. It was statistically significant when *P* value was less than 0.05.

## Results

### Patient Demographic and Clinical Characteristics

The 3,103 patients were qualified with advanced HCC (AJCC Stage III and IV) and randomized into training cohort (2,175) and validation cohort (928). The median follow-up and the interquartile range (IQR) for the whole population, the training cohort and the validation cohort were 4 months and 1–12 months, respectively. The demographic and clinical characteristics of patients with advanced HCC were presented in **Table 1**. The 1,385 patients enrolled in the study received chemotherapy and 482 patients were treated with radiotherapy. In summary, there was no statistical difference between the training cohort and validation cohort in demographic and clinical characteristics (*P* > 0.05). 

**Table 1:** Demographics and clinical characteristics of advanced HCC at diagnosis.

Variable	Whole population		Training cohort		Validation cohort		*P* value
	*n*	%	*n*	%	*n*	%	
	3,103		2,175		928		
Age year							
<65	1,878	60.52	1,308	60.14	570	61.42	0.53
>65	1,225	39.48	867	39.86	358	38.58	
Race							
Black	574	18.50	382	17.56	192	20.69	0.12
White	1,871	60.30	1,325	60.92	546	58.84	
Other	658	21.21	468	21.52	190	20.47	
Sex							
F	600	19.34	417	19.17	183	19.72	0.72
M	2,503	80.66	1,758	80.83	745	80.28	
AJCC Stages^a^							
III	1,722	55.49	1,210	55.63	512	55.17	0.81
IV	1,381	44.51	965	44.37	416	44.83	
Tumor size							
0–5 cm	1,530	49.31	1,052	48.37	478	51.51	0.20
5–10 cm	1,444	46.54	1,027	47.22	417	44.94	
>10 cm	129	4.16	96	4.41	33	3.56	
Number							
1	2,687	86.59	1,883	86.57	804	86.64	0.96
>1	416	13.41	292	13.43	124	13.36	
Regional nodes							
Negative	2,364	76.18	1,662	76.41	702	75.65	0.33
Not examined	112	3.61	84	3.86	28	3.02	
Positive	627	20.21	429	19.72	198	21.34	
Treatment							
No operation	2,819	90.85	1,987	91.36	832	89.66	0.31
Local tumor destruction	96	3.09	63	2.90	33	3.56	
Hepatectomy or transplant	188	6.06	125	5.75	63	6.79	
Radiation							
Yes	482	15.53	355	16.32	127	13.69	0.06
No	2,621	84.47	1,820	83.68	801	86.31	
Chemotherapy							
Yes	1,385	44.63	963	44.28	422	45.47	0.53
No	1,718	55.37	1,212	55.72	506	54.53	
DX bone^b^							
Yes	313	10.09	234	10.76	79	8.51	0.06
No	2,790	89.91	1,941	89.24	849	91.49	
DX lung							
Yes	397	12.79	273	12.55	124	13.36	0.55
No	2,706	87.21	1,902	87.45	807	86.96	
Marital							
Married	1,511	48.69	1,073	49.33	438	47.20	0.21
Divorced	736	23.72	497	22.85	239	25.75	
Single	856	27.59	605	27.82	251	27.05	

a AJCC Stages: The seventh edition American Joint Committee on Cancer (AJCC) TNM staging system.

b DX, distant metastasis.

### Univariate and Multivariate Cox Regression Analysis

The outcome of univariate Cox regression analysis of the training cohort revealed that age, gender, race, tumor size, tumor number, AJCC stage, bone metastasis, lymph node metastasis, lung metastasis, treatment, radiotherapy, chemotherapy, and marital status were prognostic factors for patients with advanced HCC (*P* < 0.05). Age, AJCC stage, lymph node status, tumor number, bone metastasis, lung metastasis, surgery, radiotherapy, chemotherapy, and marital status were independent prognostic factors for patients with advanced HCC (*P* < 0.05) identified in multivariate Cox regression analysis and were included in construction of the nomogram (**Table 2**).

**Table 2:** The results of univariate and multivariate Cox regression analyses on variables for the prediction of CSS of advanced hepatocellular carcinoma patients.

Variable	Univariate		*P* value	Multivariate		*P* value
	HR	95% CI		HR	95% CI	
Age						
<65	Reference			Reference		
>65	1.1	1.00–1.20	0.03	1.17	1.01–1.22	0.02
Race						
Black	Reference			Reference		
White	1.02	0.90–1.15	0.71	1.08	0.96–1.22	0.18
Other	1.09	0.95–1.26	0.19	1.25	1.07–1.45	<0.001
Sex						
F	Reference			Reference		
M	1.12	0.99–1.24	0.06	1.06	0.96–1.19	0.26
AJCC Stages^a^						
III	Reference			Reference		
IV	1.52	0.39–1.66	<0.001	1.5	1.2–1.89	<0.001
Tumor size						
0–5	Reference			Reference		
5–10	0.84	0.77–0.92	<0.05	0.93	0.85–1.03	0.19
>10	1.21	0.82–0.98	0.07	1.18	0.94–1.46	0.13
Number						
1	Reference			Reference		
>1	0.82	0.72–0.93	<0.001	0.84	0.74–0.96	0.01
Regional nodes						
Negative	Reference			Reference		
Not examined	2.08	1.67–2.60	<0.001	1.28	1.01–1.62	0.03
Positive	1.22	1.09–1.36	<0.001	1.12	0.94–1.33	0.17
Treatment						
No operation	Reference			Reference		
Local tumor destruction	0.47	0.36–0.62	<0.001	0.37	0.28–0.49	<0.001
Hepatectomy or transplant	0.31	0.25–0.38	<0.001	0.22	0.18–0.28	<0.001
Radiation						
Yes	Reference			Reference		
No	1.5	1.33–1.69	<0.001	1.93	1.70–2.10	<0.001
Chemotherapy						
Yes	Reference			Reference		
No	1.87	1.69–2.01	<0.001	2.29	2.09–2.51	<0.001
DX bone^b^						
Yes	Reference			Reference		
No	0.7	0.61–0.80	<0.001	0.78	0.66–0.93	<0.001
DX lung						
Yes	Reference			Reference		
No	0.57	0.50–0.65	<0.001	0.82	0.70–0.96	<0.001
Marital						
Married	Reference			Reference		
Divorced	1.26	1.13–1.40	<0.001	1.14	1.02–1.28	0.01
Single	1.23	1.14–1.36	<0.001	1.17	1.05–1.30	<0.001

a AJCC Stages: The seventh edition American Joint Committee on Cancer (AJCC) TNM staging system.

b DX, distant metastasis.

### Development and Validation of Nomogram

**Figure 2:**
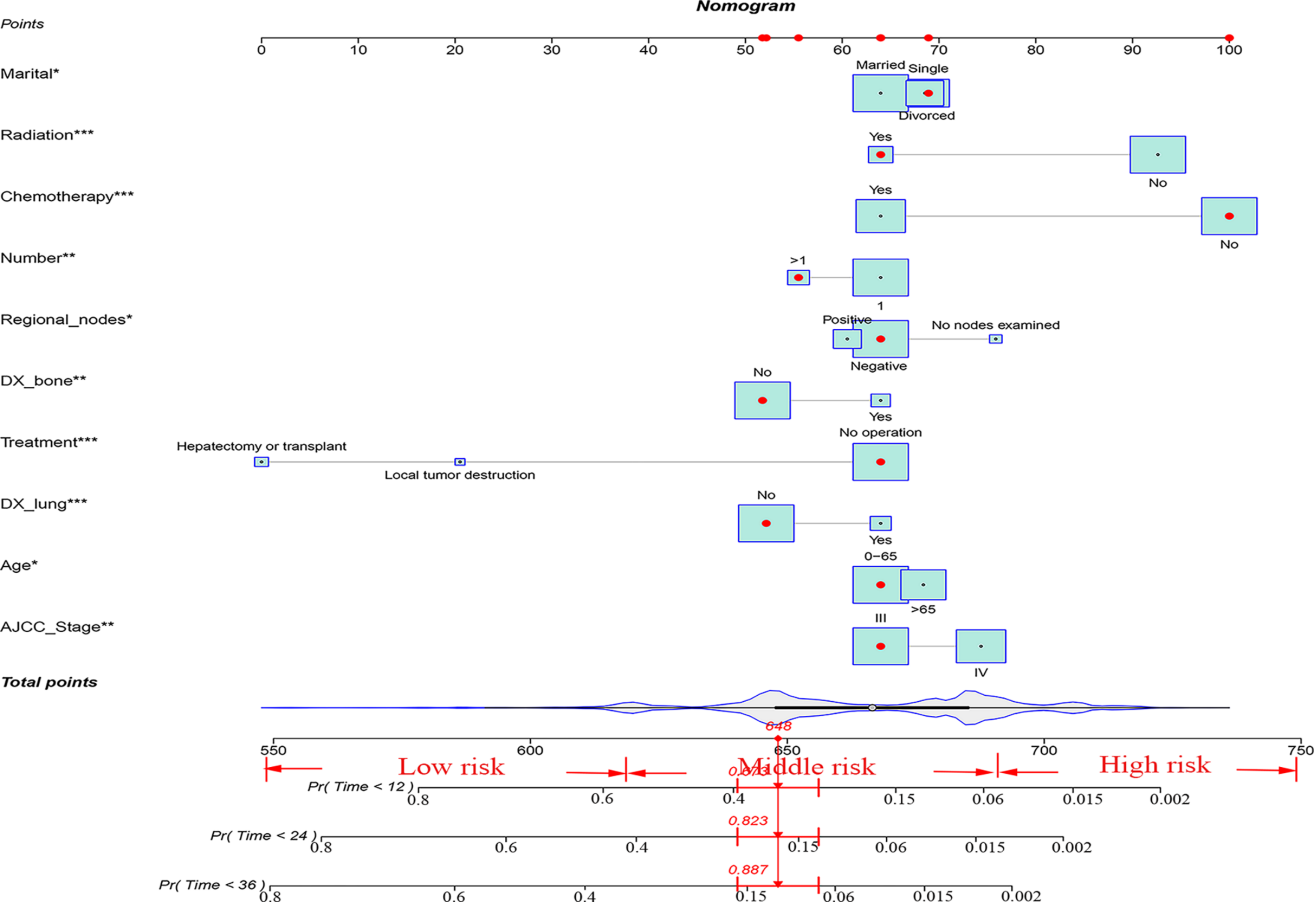
A nomogram for advanced hepatocellular carcinoma patients. *P < 0.05; **P < 0.01; ***P < 0.001.

Finally, ten variables (age, AJCC stage, lymph node metastasis, number of tumors, bone metastases, lung metastases, treatment, radiotherapy, chemotherapy, and marriage) were selected to construct the nomogram for predicting the 1-, 2-, and 3-year CSS in patients with advanced HCC (**Figure 2**). To predict the CSS for patients with advanced HCC, the score in each row of variables was found and the total score of all variables were calculated. Then located the corresponding score in the total score of the row and the 1-, 2-, and 3-year probability of CSS could be inferred by drawing a straight line on the last three rows.

**Figure 3:**
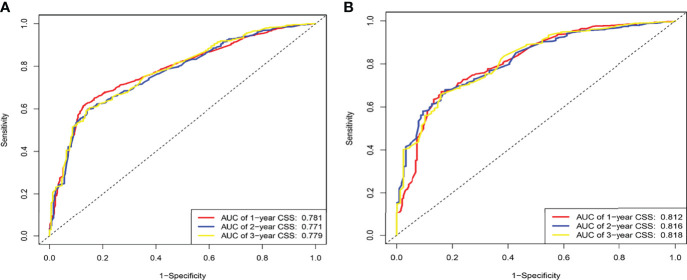
ROC of the nomogram for 1-, 2-, and 3-year prediction. **(A)** Training cohorts based on the nomogram. **(B)** Validation cohorts based on the nomogram.

**Figure 4:**
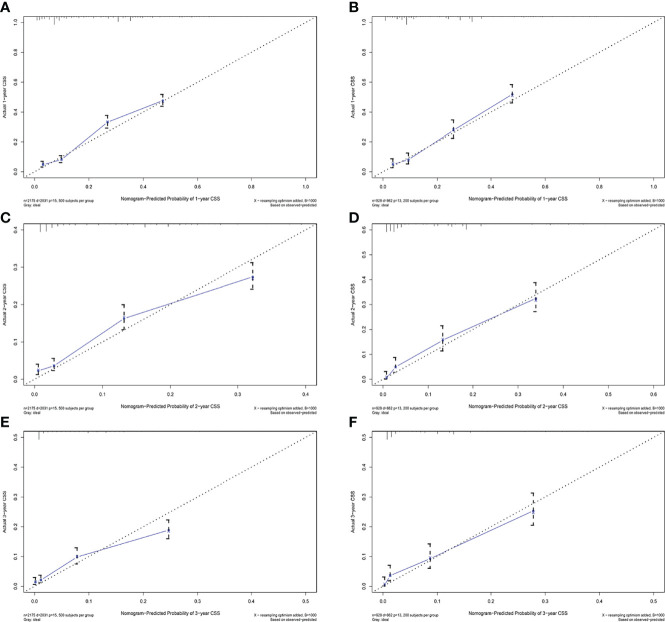
Calibration plots of 1-year, 2-year, and 3-year CSS for advanced hepatocellular carcinoma patients. **(A, C, E)** Calibration plots of 1-year, 2-year, and 3-year CSS in the training cohort. **(B, D, F)** Calibration plots of 1-year, 2-year, and 3-year CSS in the validation cohort.

**Figure 5:**
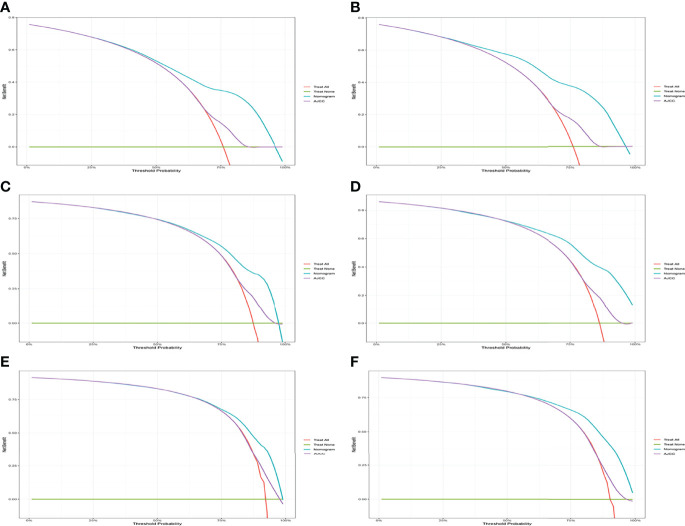
Decision curve analysis of CSS-associated nomogram and AJCC criteria. **(A, C, E)** DCA curves of 1-year, 2-year, and 3-year CSS in the training cohort. **(B, D, F)** DCA curves of 1-year, 2-year, and 3-year CSS in the validation cohort.

The C-indexes for the training and validation cohorts were 0.734 (95% CI: 0.726–0.743) and 0.732 (95% CI: 0.726–0.744), respectively. The receiver operating characteristic (ROC) curves, calibration curves, and DCA curves were shown in **Figures 3**–**5**. The analysis of the ROC curve indicated the outstanding predictive performance of the nomogram (1-, 2-, and 3-year AUC for the training cohort were 0.781, 0.771, and 0.779; and 1-, 2-, and 3-year AUC for the validation cohort were 0.812, 0.816, and 0.818). In addition, the nomogram-related DCA curves at 1, 2, and 3 years in both the training and validation cohorts revealed outstanding promising clinical application and good positive net benefit. The calibration curves all displayed a high consistency between the predicted CSS rates at 1, 2, and 3 years and the observed results.

**Figure 6:**
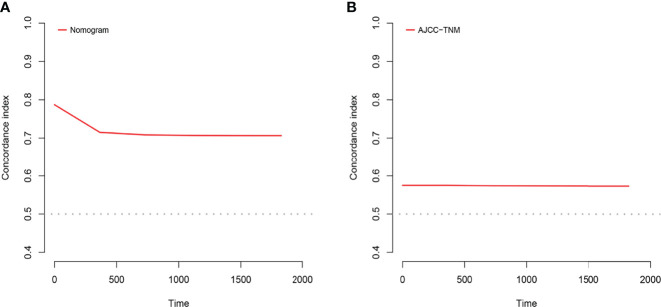
C-index analysis. **(A)** The nomogram related C-index. **(B)** AJCC staging criteria related C-index.

A comparison of the applied values of the nomogram and AJCC criteria with C-index, NRI, and IDI was performed. In the training cohort, the nomogram-related C-index was higher than that of the AJCC criteria (**Figure 6**). The 1-, 2-, and 3-year NRI were 0.77 (95% CI = 0.65–0.86), 0.46 (95% CI = 0.0.37–0.58), and 0.48 (95% CI = 0.35–0.61), respectively. The IDI values at 1, 2, and 3 years were 0.16 (95% CI 0.13–0.18, *P* < 0.001), 0.15 (95% CI 0.12–0.17, *P* < 0.001), and 0.12 (95% CI 0.09–0.16, *P* < 0.001; **Table 3**). The above results were strong enough to argue that the nomogram had a superior value of application and improved predictive capability than the AJCC stage system. In addition, the clinical benefits of columnar maps were evaluated, which was compared with those of the AJCC criteria. DCA curves in both the training and validation cohorts demonstrated that the nomogram showed better prediction for the 1-, 2-, and 3-year CSS probabilities because it produced a greater net benefit compared to the AJCC criteria and with both the treat-all-patients scheme and the treat-none scheme.

**Table 3:** NRI and IDI of the nomogram and AJCC staging criteria alone in CSS prediction for advanced hepatocellular carcinoma.

Index	Training cohort		P value	Validation cohort		P value
	Estimate	95% CI		Estimate	95% CI	
NRI						
For 1-year CSS	0.77	0.65–0.86		0.82	0.70–0.96	
For 2-year CSS	0.46	0.37–0.58		0.72	0.42–0.92	
For 3-year CSS	0.48	0.35–0.61		0.49	0.30–0.70	
IDI						
For 1-year CSS	0.16	0.13–0.18	<0.001	0.19	0.14–0.23	<0.001
For 2-year CSS	0.15	0.12–0.17	<0.001	0.18	0.12–0.22	<0.001
For 3-year CSS	0.12	0.09–0.16	<0.001	0.16	0.11–0.23	<0.001

### New Risk Stratification

**Figure 7:**
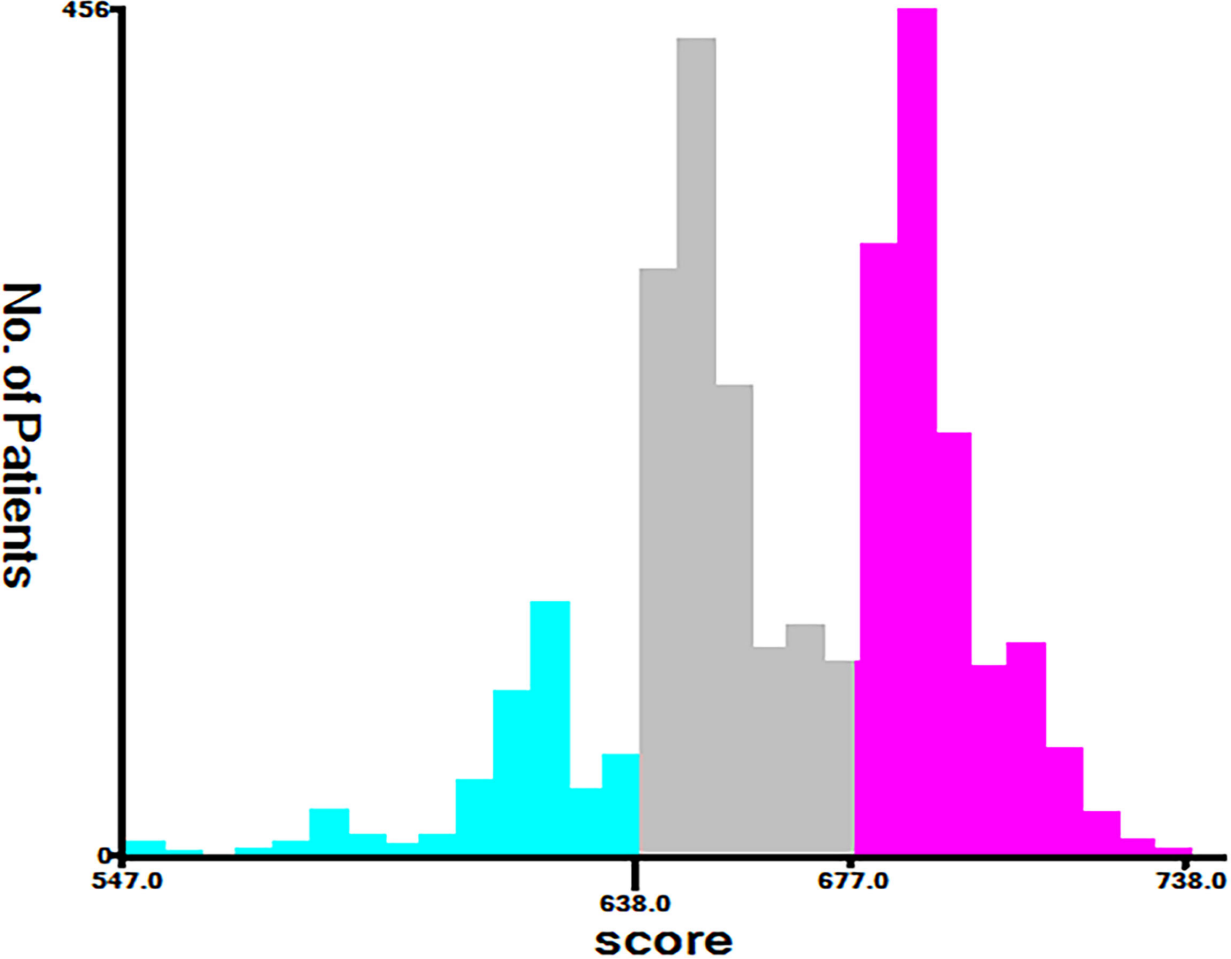
Cutoff point for risk stratifications selected using X-tile.

**Figure 8:**
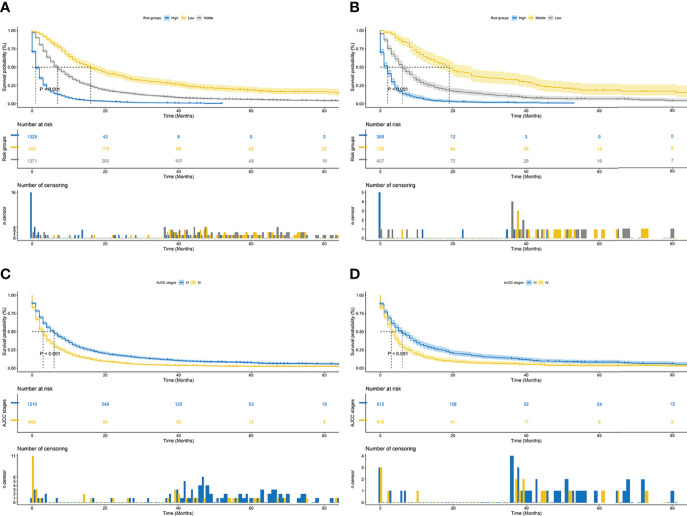
Kaplan–Meier CSS curves of patients with advanced hepatocellular carcinoma based on different criteria. **(A, B)** Kaplan–Meier CSS curves of training and validation cohorts based on the new risk stratification system. **(C, D)** Kaplan–Meier CSS curves of training and validation cohorts based on AJCC staging criteria.

Finally, risk stratification was performed by calculated with the nomogram. Patients with advanced HCC were divided into three risk groups low risk (total points < 638), middle risk (638 ≤ total points < 677) and high risk (total points ≥ 677; **Figure 7**). Kaplan–Meier curves exhibited a significant discriminatory in the three risk groups. In contrast, the AJCC criteria has shown limited ability to identify low-risk and high-risk patients in both the training cohort and validation cohort (**Figure 8**).

## Discussion

The prognosis of advanced HCC is extremely frustrating. Simultaneously, clinical prognostic models based on large cohorts are not available. Therefore, we established and validated a nomogram for predicting the prognosis of patients with advanced HCC by analyzing the data of patients obtained from the SEER database. Results of validations indicated that the nomogram had excellent predictive and discriminatory ability. Based on the nomogram, we developed a new risk stratification system for patients with advanced HCC by calculating the total score of patients (using X-tile software to select the cutoff value of the best grouping). This system divided all patients into low-, middle-, and high-risk groups. Compared with the AJCC criteria, this risk stratification has an outstanding ability to distinguish different risk groups. In addition, the system not only accurately predicts the prognosis of patients with advanced HCC, but also functions as a tool for individualized management and treatment. The significant characteristic of this study is that a new risk stratification system for patients with advanced hepatocellular carcinoma was built by applying multiple statistical methods. Based on this, the advantages and disadvantages of the new risk stratification system and AJCC staging system was explored, which were not mentioned in any other articles.

By univariate and multivariate Cox regression analysis, 10 variables (including age, AJCC stage, lymph node metastases, number of tumors, bone metastases, lung metastases, treatment, radiotherapy, chemotherapy, and marriage), which significantly affected CSS in patients with advanced HCC, and included in the nomogram. By measuring the range of scores of the incorporated variables on the nomogram score scale, treatments, lymph node metastases, chemotherapy, and radiotherapy were identified highly significant variables affecting the prognosis of patients with advanced HCC. Patients with early stage HCC did not have significant symptoms, and the majority of patients have developed advanced HCC when they were diagnosed (16). Systemic therapy was universally regarded as limited in its efficacy for patients with advanced HCC compared to other cancers (17). It was not until 2007 that sorafenib became the first drug proven to improve survival in advanced HCC. Results from several large randomized controlled trials have confirmed that compared to placebo, sorafenib prolonged the median survival time of patients with advanced HCC (18, 19), which was consistent with our findings. Recently, Llovet et al. (7) demonstrated that sorafenib combined with immunotherapy was superior to single agent efficacy, and the new findings were expected to improve the treatment paradigm for patients with advanced HCC (20–22). Local therapy was a bridge between liver transplantation and hepatectomy and was also the primary treatment modality for advanced HCC. Hanje et al. (23) reported 4-year survival rate was 92% for patients treated with liver transplantation after reaching Milan criteria at the descending stage. Salem et al. (24) reported an objective remission rate of 42% in patients treated locally. Although studies have confirmed the potential value of radiotherapy in specific patients with HCC (25, 26), especially in patients with early stage who were unable to be treated with transplantation or resection. However, the efficacy of radiotherapy in the treatment of advanced HCC remained controversial (27). Patients with advanced HCC were less likely to be tested for lymph node metastasis because of the inability to undergo surgery. Therefore, the prognostic effects of lymph node metastasis on patients with advanced HCC remained to be determined.

Tumor staging based on AJCC criteria was the predominant option for predicting prognosis in patients with advanced HCC. However, the effects of age, treatment, marital status, and other variables on patient prognosis were not considered in the traditional AJCC-based criteria (28, 29). We synthesized multiple variables affecting CSS in patients with advanced HCC (including demographic and clinicopathologic characteristics) into a nomogram. In addition, patients were divided into low-, middle-, and high-risk groups based on their total scores. On this basis, the power of the nomogram and the traditional AJCC-based criteria were compared, which other articles have not explored. The results of NRI, IDI, and C-index indicated that the nomogram had improved predictive power over tumor staging based on AJCC criteria alone. In addition, DCA demonstrated the clinical benefit and utility of our nomograms in predicting CSS over conventional staging systems. Remarkably, Kaplan–Meier analysis displayed significantly distinct CSS among the three risk groups, with considerably discriminatory power than the conventional staging system. In particular, the nomogram had a higher ability to distinguish between high-risk and low-risk groups than the traditional staging system, which can assist clinicians in individualizing the treatment and management.

Although the nomogram demonstrates outstanding utility, this study still has certain limitations. For example, the SEER database did not collected hematological indicators of patients, which therefore were not included in the screening. In addition, this study assessed these variables despite internal validation; our model lacks a multicenter clinical sample to perform external validation so as to provide more convincing evidence.

## Conclusion

In conclusion, the nomogram exhibits powerful predictive performance, superior clinical benefit, and accurate predictive efficacy compared to the AJCC staging system. It can be applied to predict CSS in patients with advanced hepatocellular carcinoma.

## Data Availability Statement

The datasets presented in this study can be found in online repositories. The names of the repository/repositories and accession number(s) can be found in the article/supplementary material. 

## Ethics Statement

The SEER database was publicly available and the private data of all patients have been eliminated from the SEER database. Therefore, informed consent and institutional review board approval were not required.

## Author Contributions

Conceptualization: DY, YS, and FZ. Data curation: CC and KZ. Formal analysis: DY, XX, and YS. Writing—original draft: DY, YS, and FZ. Writing—review and editing: YD. All authors contributed to the article and approved the submitted version.

## Conflict of Interest

The authors declare that the research was conducted in the absence of any commercial or financial relationships that could be construed as a potential conflict of interest.

## Publisher’s Note

All claims expressed in this article are solely those of the authors and do not necessarily represent those of their affiliated organizations, or those of the publisher, the editors and the reviewers. Any product that may be evaluated in this article, or claim that may be made by its manufacturer, is not guaranteed or endorsed by the publisher.
